# Biophysical stimulation in osteonecrosis of the femoral head

**DOI:** 10.4103/0019-5413.45319

**Published:** 2009

**Authors:** Massari Leo, Fini Milena, Cadossi Ruggero, Setti Stefania, Traina GianCarlo

**Affiliations:** Department of Biomedical Sciences and Advanced Therapies, Orthopaedic Clinic, University of Ferrara, Italy; 1 Laboratory of Experimental Surgery, Rizzoli Orthopaedic Institute, Bologna, Italy; 2 IGEA, Clinical Biophysics, Carpi, Modena, Italy

**Keywords:** Chondroprotective effect, osteonecrosis of the femoral head, pulsed electromagnetic fields

## Abstract

Osteonecrosis of the femoral head is the endpoint of a disease process that results from insufficient blood flow and bone-tissue necrosis, leading to joint instability, collapse of the femoral head, arthritis of the joint, and total hip replacement. Pain is the most frequent clinical symptom. Both bone tissue and cartilage suffer when osteonecrosis of the femoral head develops. Stimulation with pulsed electromagnetic fields (PEMFs) has been shown to be useful for enhancing bone repair and for exerting a chondroprotective effect on articular cartilage. Two Italian studies on the treatment of avascular necrosis of the femoral head with PEMFs were presented in this review. In the first study, 68 patients suffering from avascular necrosis of the femoral head were treated with PEMFs in combination with core decompression and autologous bone grafts. The second one is a retrospective analysis of the results of treatment with PEMFs of 76 hips in 66 patients with osteonecrosis of the femoral head. In both studies clinical information and diagnostic imaging were collected at the beginning of the treatment and at the time of follow up. Statistical analysis was performed using chi-square test. Both authors hypothesize that the short-term effect of PEMF stimulation may be to protect the articular cartilage from the catabolic effect of inflammation and subchondral bone-marrow edema. The long-term effect of PEMF stimulation may be to promote osteogenic activity at the necrotic area and prevent trabecular fracture and subchondral bone collapse. PEMF stimulation represents an important therapeutic opportunity to resolve the Ficat stage-I or II disease or at least to delay the time until joint replacement becomes necessary.

## INTRODUCTION

Osteonecrosis is a common disorder of bone circulation and it is particularly common in the femoral head; there are an estimated 20,000–30,000 new cases diagnosed annually.^1^ The syndrome may be minor, nonprogressive, asymptomatic, or it may result in the destruction of subchondral cortical bone leading to major problem for the hip.

There are two types of pathologic osteonecrotic disorders: medullary and corticocancellous involving the joint.^2^ Medullary osteonecrosis is caused by interference in the blood supply to the medullary cavity, which results in the death of the cells of the trabecular bone. Corticocancellous osteonecrosis involving the joint is usually much more pernicious and causes much more patients distress; the vascular compromise usually occurs in the proximal femur, and both the trabecular and the subchondral bone die and the region does not calcify as in the medullary disease.

Pathogenesis of osteonecrosis refers to all the cellular events, reaction, and other pathological mechanisms occurring in the development and progression of the disease. There are many theories of the pathogenesis of osteonecrosis of the femoral head. Some authors postulated four possible structural or biological disorders by which the blood supply is interrupted: mechanical interruption, vascular thrombosis or embolism, external injury or pressure on a vascular wall, and venous occlusion. Most recently, some authors hypothesized that an impaired mesenchymal cellular differentiation may be a potential mechanism of action of the pathogenesis of osteonecrosis.^3^ Arterial occlusion seems to be the main contributing factor. The two main pathologic mechanisms are thrombotic and embolic. Arterial thrombosis can occur in two different ways that can act synergistically. There may be a primary injury of the arterial wall or a primary coagulation disorder.^4^

Several authors have described a thrombosis caused by an injury of the endothelial layer by arteriosclerosis or other occlusive vascular disorders.^5^–^7^ In healthy subjects there is a controlled balance between thrombosis and/or hypofibrinolysis; the presence of hereditary or acquired thrombophilia and/or hypofibrinolysis can modify this balance leading to a thrombotic event.^8^

Another possible cause for avascular necrosis of the femoral head is the embolic disorder. An alteration of lipid metabolism causes an increase in circulating lipids due to a reduced lipolytic enzymatic activity. Circulating lipids accumulate in the liver causing fatty degeneration and adipose cyst formation. Rupture of the cysts or spontaneous diffusion of droplets from the liver leads to the formation of fat emboli in the bloodstream. From the pulmonary filter they can reach any other visceral site or bone. The subchondral region of the femoral head is an area of localization of fat emboli due to the small diameter and to terminal feature of arterial vessel at this level. Intraosseous fat emboli cause stasis through mechanical occlusion and also endothelial damage following their hydrolysis to free fatty acids. Another mechanism involved in the pathogenesis is an external compression of capillaries by the hypertrophy of marrow fat and cells with an increase of intramedullary pressure and reduction of blood flow.^9^,^10^

There are also some other disorders associated with osteonecrosis, such as AIDS, alcoholism, aging, corticosteroid administration, diabetes, fracture and dislocation, smoking status, obesity, and rheumatoid arthritis. Some authors currently believe that the most frequent cause is the use of corticosteroids, which induces a reduction in the concentration of a lipoproteic lipase, with a consequent increase in concentrations of fat.^11^

## TREATMENT STRATEGIES AND CLINICAL RESULTS

Retrospective clinical studies show that osteonecrosis of the femoral head in 80–90% of affected patients inevitably progresses to destroy the femur head, usually within 2–3 years of diagnosis.^12^ Osteonecrosis of the femoral head is a devastating disease that typically affects young patients (mean age, mid-thirties) and often leads to femoral head collapse if left untreated. Because these patients are young and relatively active, and because total hip arthroplasty has not always been associated with an optimal outcome in this patient population, various procedures for joint preservation in the early stages of the disease have been utilized, ranging from core decompression, percutaneous drilling, vascularized and nonvascularized bone-grafting, and osteotomies to hemiresurfacing. However, the success rate of these joint preserving operations are variable, and long- term success is not always reliable.^13^,^14^ In advanced osteonecrosis of the femoral head, total hip arthroplasty and metal-on-metal total hip resurfacing is necessary to provide joint functional recovery and pain relief. The National Health Insurance Bureau declared that the percentage of patients receiving total hip arthroplasty for nontraumatic osteonecrosis of the femoral head is more than 50% in Taiwan, Japan, and Korea; it is 2.2% in Sweden (85% for degenerative or traumatic arthritis), and 12% of 30,000 cases occur annually in the United States for nontraumatic osteonecrosis of the femoral head.^15^ The patients undergoing arthroplasty can be expected to require multiple future surgeries and should be well educated in this regards before surgery.

Alternative nonoperative treatments that conserve bone or that promote osteogenesis and angiogenesis as well as treatment that heal subchondral bone fractures should be taken into consideration. A treatment of the modern orthopedic surgery is represented by the regulation of skeletal repair by biophysical agents, including mechanical strain, ultrasound, and pulsed electromagnetic fields (PEMFs). Several double-blinded and controlled clinical trials have been conducted to demonstrate the clinical effectiveness of PEMFs to stimulate osteogenetic activity in fresh fractures, osteotomies, nonunions, spine fusion, and ultimately in the osteonecrosis of the femoral head in isolation or in combination with surgical techniques.^16^–^21^

In Italy, two different studies were conducted on the use of biophysical stimulation with PEMFs for the treatment of avascular necrosis of the femoral head.

**Figure 1:**
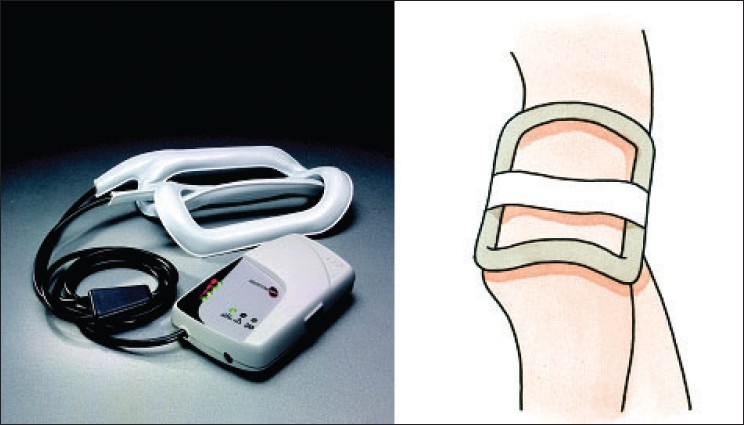
Left: Clinical pulsed electromagnetic field generator; Right: Schematic representation of treatment modality

In the first Italian study, 68 patients suffering from avascular necrosis of the femoral head were treated with PEMFs for 8 hours per day for six months, in combination with core decompression and autologous bone grafts, obtained from proximal metaphysis area and femoral neck.^22^ The pulse generator (SPT BIOSTIM; IGEA, Carpi, Italy [Figure 1]) supplied the coil with single-voltage pulses at 75 Hz, with each pulse of 1.3 ms. Patients were classified according the Steinberg staging by standard radiographs [Table 1]; magnetic resonance images and scintigrams were acquired to confirm the diagnosis. The average age was 44 years. The etiology of the necrosis was primary (n=34); corticosteroid therapy (n=17); and secondary to trauma (n=5). The mean duration of follow-up was 5.8 years (3 months to 11 years). To evaluate the bone grafts integration, instrumental evaluations (Rx and RMN) were performed at each follow-up: 1, 3, 6, 12, and 24 months from surgery. Then, radiographic evaluations were conducted each year and magnetic resonance every two or three years as requested by the surgeon.

**Table 1:** Avascular necrosis staging

Steinberg stage	Patients
I	28
II	24
III	11
IV	5

In 81% of patients with Steinberg stage -II, no pain and limping was detected and good radiographic results were reported. In patients with Steinberg stage -III, the success rate was reduced to 70%, and in patients with stage -IV, the good clinical and radiographic results were 53% and 27%, respectively. Total hip arthroplasty was necessary only in two patients (bilaterally in one patient with Steinberg stage -III and in one hip with Steinberg stage -IV).

**Figure 2:**
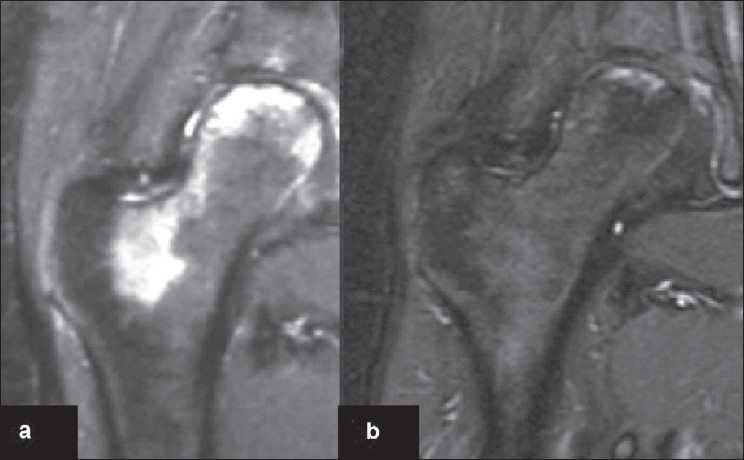
MRI of right hip (a) of 17 year old female shows edema with ficat stage II osteonecrosis shows bone edema. (b) MRI of the same hip after 6 months of stimulation and at the end of the treatment shows decreased bone edema and the patient had no pain.

The second Italian study is a retrospective review of 66 consecutive patients (76 hips) who received PEMFs (SPT BIOSTIM; IGEA, Carpi, Italy) for the treatment of Ficat stage-I, II, or III osteonecrosis of the femoral head.^23^ In this study, there were no identifiable associated risk factors in 51 patients (77%), whereas a predisposing factor could be identified in 15 patients (23%). The primary endpoint analyzed in this study was the avoidance of hip surgery, and the secondary endpoint was limiting the radiographic progression of osteonecrosis of the femoral head. Treatment with PEMFs was started at the time of the initial diagnosis and the patients were instructed to use the stimulator for 8 hours per day for six months. The mean duration of treatment was 5 ± 2 months. At the time of final follow-up, (a mean of 28 months), PEMFs preserved 50 (94%) of the 53 Ficat stage-I or -II hips [Figure 2]; the authors found 15 of the 76 hips (20%) had progression of osteonecrosis of the femoral head that led to the onset of a severe degenerative osteoarthritis and required surgery. Of these 15 hips, 12 had Ficat stage-III disease at the beginning of treatment, whereas the remaining three hips had Ficat stage-II disease at the beginning of treatment. All the patients reported with pain at the start of the treatment were pain free after 60 days of stimulation in 35 patients (53%) and pain was of moderate intensity in 17 patients (26%).

## DISCUSSION

Bassett.^24^ (1989) was the first to propose PEMFs in treating 118 patients with avascular necrosis of the femur head, Steinberg assessed the possibility of employing electrical stimulation by faradic systems (implanted electrodes) and, subsequently, the feasibility of using capacitive systems.^25^,^26^ In both studies the stimulation was performed in combination with core decompression (CD); although the results obtained were good, it was not possible to demonstrate any statistically significant advantage of the combined treatment as against treatment with CD alone. Aaron performed a series of clinical studies aimed to evaluate the treatment of AVN with CD and PEMFs, whether used separately or in association; 657 hips underwent treatment.^27^ The hips have been studied with a follow-up of 36 months. The results of the stimulation with PEMFs were compared with those obtained by CD and CD + PEMFs in stage -II and stage -III hips. In stage -II, PEMFs and PEMFs + CD gave better results than CD alone. From the radiographic point of view, however, CD + PEMFs gave better results than PEMFs alone and CD. In the clinical outcomes, that PEMFs and CD + PEMFs gave greater probability of survival than CD alone.

The Italian experiences exhibits the typical features and shortcoming of not controlled study but the findings reported here confirm those previously obtained by other authors.^28^,^29^ The results of the studies suggest that an early instrumental diagnosis at the onset of the disorder and the immediate use of PEMFs enables better clinical results. The authors observed that the lesions in early stage respond well to treatment with PEMFs and, in the majority of cases, as far as Ficat II, are capable of preventing the progression of the disease. Ficat stage -III does not constitute a real indication for this treatment and must always be carefully assessed, the treatment eventually being advised for the younger patients. Idiopathic lesions seem more sensitive to PEMF therapy as compared with secondary forms, because the therapies responsible of the disease cannot be interrupted. The weight bearing does not influence the evolution of lesion. This observation is unexpected, but it has also been highlighted in other studies. The relief from pain accompanied by recovery of the joint function is important, as observed in the majority of the patients within 45 days from beginning of treatment. It is likely that this effect is mediated by the antiinflammatory action of the PEMFs that enables resolution of the joint edema. With regard to this, both authors concluded that in the presence of avascular necrosis of the femoral head we can distinguish two different aspects: one concerning the cartilage and one concerning the subchondral bone tissue.

The initial stages of the disease are dominated by the ischemic and inflammatory component; the latter is responsible for the joint pain, edema, and degradation of the cartilage. In 2002, some authors have demonstrated that the positive strong antiinflammatory effects of PEMFs causes an increase of adenylyl cyclase activity and a reduction of superoxide anion production, as a result of upregulation of the A_2A_ receptors located on the neutrophil surface.^30^ Recently, this antiinflammatory action has been demonstrated on the sinoviocytes and chondrocytes.^31^ The binding plays a fundamental role on the inflammatory response and promotes new vessel formation and limits the extension of the necrotic area, resulting from ischemia. This effect is of extreme importance because of the role of the synovial membrane on the revascularization and resorption of necrotic bone. Moreover, biophysical stimulation is able to inhibit the catabolic effects of the inflammatory cytokines on the joint cartilage^32^ and to stimulate the synthesis of proteoglycans and the local production of BMPs, TGF-α, and IGF-I;^33^ PEMFs exert a chondroprotective effect *in vivo* on osteoarthritis,^34^ which may play a fundamental role in the treatment of early stages of osteonecrosis of the femoral head by limiting the damage induced by inflammation and preserving cartilage and subchondral bone exerting a short term effects.

Regarding the bone tissue, in the long term, the main problem to resolve is the avascular deficit, the first step to get to the necrosis and the recovery of the ischemic bone tissue. In this condition, the PEMF has effects firstly on angiogenesis and then on osteogenesis. In association with core decompression, biophysical stimulation enhances the osteointegration of autologous bone grafts,^35^ stimulates the local production of growth factors, and favors the osteogenic activity of osteoblasts reducing the bone reabsorption by interfering with the activation of the parathyroid hormone receptor, thus decreasing the recruitment and differentiation of osteoclasts.^36^–^38^

In conclusion, both authors suggest that the treatment with PEMFs by now represents one of the conservative therapies used in the first stages of avascular necrosis of the femoral head at Ficat 0, I, II or Steinberg II and III but require from patients time of treatment, care, and precision to ensure the success expected by physicians and patients. The stimulation with PEMFs may be a good opportunity to resolve the disease or at least to delay the time until joint replacement becomes necessary.
